# Retrospective analysis of complications in 190 mandibular resections and simultaneous reconstructions with free fibula flap, iliac crest flap or reconstruction plate: a comparative single centre study

**DOI:** 10.1007/s00784-020-03607-8

**Published:** 2020-10-06

**Authors:** Lucas M. Ritschl, Thomas Mücke, Diandra Hart, Tobias Unterhuber, Victoria Kehl, Klaus-Dietrich Wolff, Andreas M. Fichter

**Affiliations:** 1 Department of Oral and Maxillofacial Surgery, School of Medicine, Technical University of Munich, Klinikum rechts der Isar, Ismaninger Straße 22, 81675 Munich, Germany; 2 Department of Oral and Maxillofacial Surgery, Malteser Kliniken Rhein-Ruhr, Kurfürstenstraße 69, 47829 Krefeld-Uerdingen, Germany; 3 School of Medicine, Institute of Medical Informatics, Statistics and Epidemiology, Technical University of Munich, Grillparzerstraße 18, 81675 Munich, Germany

**Keywords:** Mandibular reconstruction, Free bone flaps, Free fibula flap, Complications, Radiation

## Abstract

**Objectives:**

The purpose of this study was to evaluate the incidence of complications following mandibular reconstruction and to analyse possible contributing factors.

**Materials and methods:**

Clinical data and computed tomography scans of all patients who needed a mandibular reconstruction with a reconstruction plate, free fibula flap (FFF) or iliac crest (DCIA) flap between August 2010 and August 2015 were retrospectively analysed.

**Results:**

One hundred and ninety patients were enrolled, encompassing 77 reconstructions with reconstruction plate, 89 reconstructions with FFF and 24 reconstructions with DCIA flaps. Cutaneous perforation was most frequently detected in the plate subgroup within the early interval and overall (each *p* = 0.004). Low body mass index (BMI) and total radiation dosage were the most relevant risk factors for the development of analysed complications.

**Conclusions:**

Microvascular bone flaps have overall less skin perforation than reconstruction plates. BMI and expected total radiation dosage have to be respected in choice of reconstructive technique.

**Clinical relevance:**

A treatment algorithm for mandibular reconstructions on the basis of our results is presented.

**Electronic supplementary material:**

The online version of this article (10.1007/s00784-020-03607-8) contains supplementary material, which is available to authorized users.

## Introduction

Mandibular reconstruction after partial or continuity resection is a complex procedure with non-negligible sequela on the oro-pharyngeal integrity and function, aesthetics and quality of life [1–3]. With continuous technical and surgical improvements, the restoration of mandibular continuity has become a highly standardized procedure in high-volume centres over the last decades. Microvascular tissue transplantation has evolved to a gold standard procedure in reconstructive cranio-maxillofacial surgery, especially since the introduction of the free fibula flap (FFF) for mandibular reconstruction by Hidalgo [4, 5]. Another milestone in the continuous development and improvement of mandibular reconstruction was the introduction of computer-aided design and computer-aided manufacturing (CAD/CAM) in this field. The application of the CAD/CAM technique increased the quality, function, safety and symmetry of this surgical procedure and reduced the operating time significantly [2, 6–8].

Microvascular bone transplants like the FFF or iliac crest (DCIA) flap are reported to be safe reconstructive possibilities with transplant-specific advantages and disadvantages. In general, they are associated with good long-term results in specialized centres and have a reliable consistency of vertical bone dimension in conventional panoramic radiographs [9, 10].

Nevertheless, mandibular reconstruction is also associated with complications, which might vary according to the reconstructive technique applied and the demanding patient cohort itself [11]. Postoperative complications after reconstructive microsurgery are generally associated with prolonged in-hospital stay, increased costs and prolonged treatment in the outpatient setting. This potentially reduces the quality of life in the synopsis.

The purpose of this retrospective study was to evaluate short- and intermediate-term complications and to describe possible factors that might contribute to the unwanted clinical course.

## Materials and methods

### Ethical statement and patient recruitment

All clinical investigations and procedures were conducted according to the principles expressed in the Declaration of Helsinki. The retrospective analysis was approved by the Ethical Committee of the local government based on this informed consent to participate in this study and on the willingness to undergo the required medical care (Approval No. 87/17 S).

Any patient requiring a mandibulectomy and simultaneous reconstruction with a reconstruction plate (intraoperatively or preoperatively bend), a FFF (conventional free hand or CAD/CAM-assisted) or a DCIA flap because of a benign or malign process between August 2010 and August 2015 at our department was included in this retrospective study. Patients with a history of previous microvascular flap loss or reconstruction with a scapula free flap and patients receiving more than one flap (double free or sandwich flap reconstruction) in the same operation were excluded [12, 13]. No further exclusion criteria were applied.

### Patient collective and study analyses

Patient records and datasets of all patients operated with a standardized scheme for mandibular continuity resection between August 2010 and August 2015 were screened for postoperative complications including revision of microvascular anastomosis and (partial) flap loss in microvascular free flap cases. Postoperative fistula, dehiscence, intraoral bone exposure, cutaneous perforation, screw loosening and plate fracture were registered in all enrolled cases. Fistula was defined as an extraoral, round wound with a diameter < 10 mm, not necessarily adjacent to the incision line/scar communicating with the oral cavity or with an abscess formation. Dehiscence was defined as an extraoral wound adjacent to the former incision line/scar with a longitudinal extent of > 10 mm.

The incidence of any complication was registered, including the time interval between operation and date of recorded incidence. We defined three clinical observation intervals: early (≤ 100 days) and late complication (> 100 days) as well as overall complication.

Potential contributing factors for the registered complications like age, gender, body mass index (BMI), ASA status, history of smoking, diabetes mellitus and history of chemo- and radiation therapy were additionally recorded.

### Statistical methods

The occurrence of complications was described by treatment group using absolute and relative frequencies. Hypothesis testing of differences between subgroups was performed by *χ*^2^ or Fisher’s exact test as necessary. Continuous variables were summarized by reconstruction type with mean and standard deviation and compared between groups using the Mann-Whitney *U* test.

All statistical tests were performed on an exploratory two-sided 5% significance level. No adjustment for multiple testing was incorporated. Analysis was done with IBM SPSS 24 for Windows software (IBM Corp, Armonk, NY).

## Results

### Clinical outcome and descriptive results (Tables 1, 2 and Figure 1)

One hundred and ninety patients were enrolled for final analysis. The overall distribution of gender, age, indication, history of surgical intervention and radiation for the reconstructive subgroups is shown in Table 1. Mandibles were reconstructed with reconstruction plates in 77 cases, FFF in 89 cases and DCIA flap in 24 cases after continuity resection. In cases with reconstruction plates, 37 plates were bent intraoperatively, and 40 plates were individually pre-bend using an in-house printed mandible (ProJet 160 printer, VisiJet PXLCore Eco and Binder; 4D Concepts GmbH, Gross-Gerau, Germany) as described by others [14]. In the case of FFF, 63 cases were reconstructed conventionally by free hand, and 26 reconstructions were performed using the CAD/CAM technique as described elsewhere [15, 16].

**Table 1:** Overview of enrolled patients and their reconstruction technique with regard to gender, indication, history of surgical therapy and radiation therapy

	Rec. plate intraop. bend (*n* = 37)	Rec. plate pre-bend (*n* = 40)	FFF conventional (*n* = 63)	FFF CAD/CAM (*n* = 26)	DCIA flap (*n* = 24)
Gender male (%)	21 (56.8)	25 (62.5)	41 (65.1)	18 (69.2)	13 (54.2)
Age median (range)	62.5 (43–86)	66.0 (14–93)	60.0 (27–76)	56.0 (18–76)	53.5 (24–71)
Malignancy yes (%)	29 (78.4)	29 (72.5)	27 (42.9)	10 (38.5)	2 (8.3)
1^st^ surgical intervention Yes (%)	25 (67.6)	30 (75.0)	33 (52.4)	11 (42.3)	15 (62.5)
History of radiation Yes (%)	13 (35.1)	9 (22.5)	40 (63.5)	13 (50.0)	2 (8.3)
Adjuvant radiation Yes (%)	18 (48.6)	17 (42.5)	13 (20.6)	5 (19.2)	2 (8.3)

*Rec.* reconstruction, *intraop.* intraoperatively, *FFF* free fibula flap, *CAD/CAM* computer-aided design and computer-aided manufacturing, *DCIA* deep circumflex iliac artery

**Table 2:** Analysis of postoperative incidence of most common complications

Complication	Rec. plate intraop. bend *n* (%)	Rec. plate pre-bend *n* (%)	FFF conventional *n* (%)	FFF CAD/CAM *n* (%)	DCIA flap *n* (%)	*p* value Fisher exact test
Revision anastomosis ≤ 30 d	/	/	6 (9.5)	4 (15.4)	0 (0.0)	0.137
Flap failure ≤ 30 d	/	/	2 (3.2)	2 (7.7)	0 (0.0)	0.502
Partial flap loss ≤ 30 d	/	/	3 (4.8)	1 (3.8)	0 (0.0)	1.000
Fistula overall	6 (16.2)	6 (15.0)	14 (22.2)	4 (15.4)	4 (16.7)	0.920
Fistula ≤ 100th d	3 (8.1)	4 (10.0)	3 (4.8)	1 (3.8)	2 (8.3)	0.791
Fistula > 100th d	3 (8.1)	2 (5.0)	11 (17.5)	3 (11.5)	2 (8.3)	0.401
Dehiscence overall	10 (27.0)	8 (20.0)	13 (20.6)	3 (11.5)	2 (8.3)	0.378
Dehiscence ≤ 100th d	8 (21.6)	5 (12.5)	9 (14.3)	2 (7.7)	0 (0.0)	0.121
Dehiscence > 100th d	2 (5.4)	1 (2.5)	2 (3.2)	1 (3.8)	1 (4.2)	0.955
Io bone exposure overall	3 (8.1)	2 (5.0)	5 (7.9)	1 (3.8)	1 (4.2)	0.938
Io bone exposure ≤ 100th d	1 (2.7)	0 (0.0)	2 (3.2)	1 (3.8)	0 (0.0)	0.818
Io bone exposure > 100th d	2 (5.4)	2 (5.0)	4 (6.3)	0 (0.0)	1 (4.2)	0.854
Cutaneous perforation overall	11 (29.7)	3 (7.5)	4 (6.3)	1 (3.8)	1 (4.2)	0.004*
Cutaneous perforation ≤ 100th d	5 (13.5)	1 (2.5)	0 (0.0)	0 (0.0)	0 (0.0)	0.004*
Cutaneous perforation > 100th d	5 (13.5)	2 (5.0)	4 (6.3)	1 (3.8)	1 (4.2)	0.617
Screw loosening overall	2 (5.4)	2 (5.0)	2 (3.2)	2 (7.7)	2 (8.3)	0.566
Screw loosening ≤ 100th d	0 (0.0)	0 (0.0)	0 (0.0)	0 (0.0)	1 (4.2)	0.004*
Screw loosening > 100th d	2 (5.4)	2 (5.0)	2 (3.2)	2 (7.7)	1 (4.2)	0.972
Plate fracture overall	0 (0.0)	2 (5.0)	3 (4.8)	4 (15.4)	0 (0.0)	0.067
Plate fracture ≤ 100th d	0 (0.0)	0 (0.0)	1 (1.6)	2 (7.7)	0 (0.0)	0.172
Plate fracture > 100th d	0 (0.0)	2 (5.0)	2 (3.2)	2 (7.7)	0 (0.0)	0.371

*d* days, *Rec.* reconstruction, *intraop.* intraoperatively, *FFF* free fibula flap, *DCIA flap* deep circumflex iliac artery, *CAD/CAM* computer-aided design/computer-aided manufacturing, *Io* intraoral

*Significant at the 5% level

**Fig. 1:**
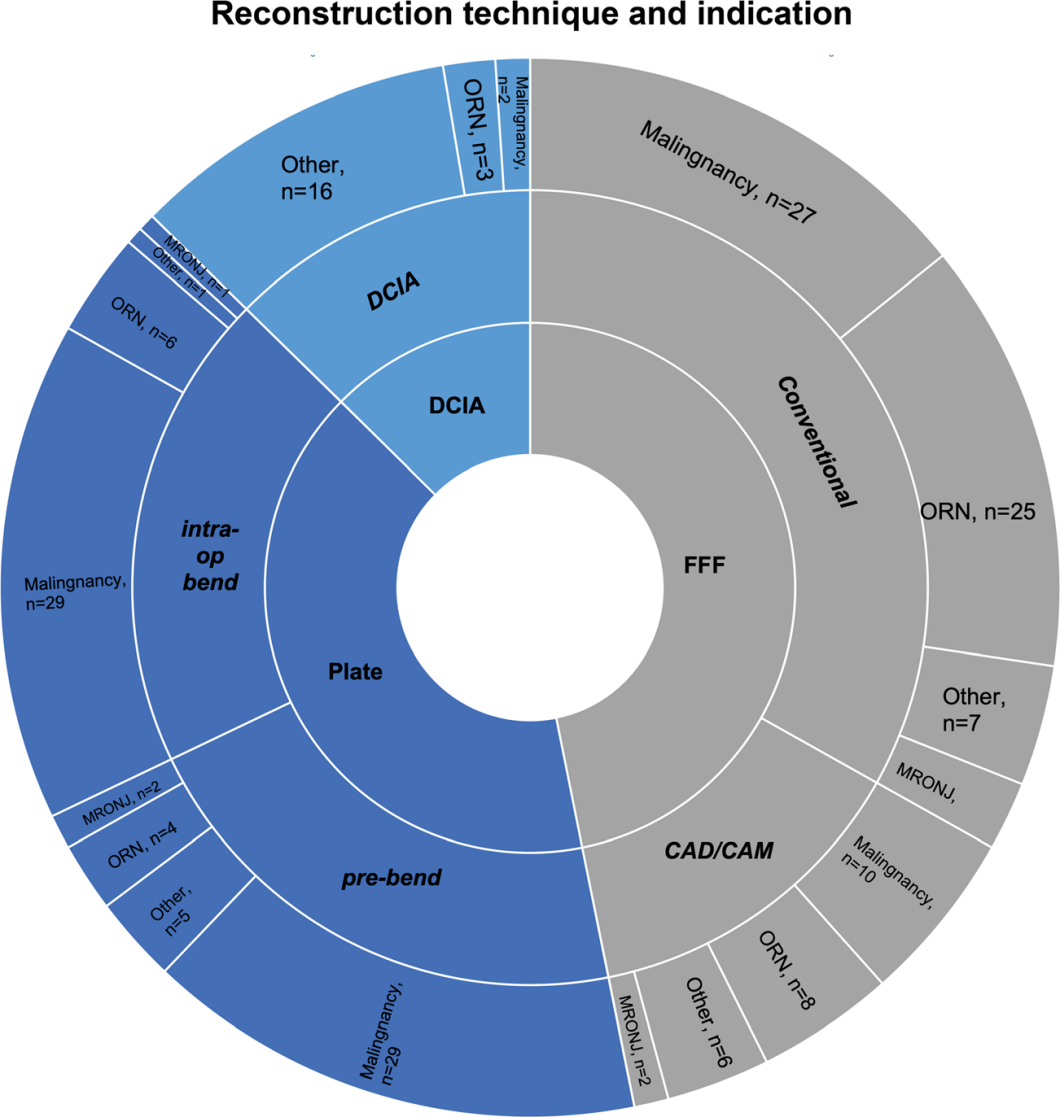
Sunburst diagram with the distribution of reconstruction techniques according to the underlying indication for mandibular reconstruction (FFF, free fibula flap; DCIA, deep circumflex iliac artery; CAD/CAM, computer-aided design and computer-aided manufacturing; ORN, osteoradionecrosis; MRONJ, medication-related necrosis of the jaw)

Median overall follow-up time was 1728 days (8–3016) for reconstructions plate, 1794 days (23–3060) for FFF and 2017 days (1070–2792) for DCIA. More precisely it was 1728 days (359–3016) for intraoperatively bend and 1726 days (8–2973) pre-bend plates and 2039 days (23–3060) for conventional FFF and 1407 days (181–2015) for CAD/CAM FFF.

Incidence and distribution of all registered complications are listed in Table 2. Ten anastomoses had to be revised in the FFF reconstructed cases (six cases conventional FFF and four CAD/CAM FFF), which resulted in four total flap losses (two cases each in the conventional and CAD/CAM FFF groups) and four partial losses of the skin paddle (three cases conventional FFF and one CAD/CAM FFF) in total. Overall, the median time of revision of failing FFF was 5 days (range 1–21), and final flap loss was seen at 17 days (6–29). No DCIA flap required revision and no flap failure occurred within the retrospective observation time.

Postoperative fistula occurred four times in the reconstruction plate group, seven time in the plate, four times in the FFF and twice in the DCIA reconstructed cases within 100 days (*p* = 0.791). Within the late interval (> 100 days), fistula occurred five times in the plate group, 14 times the FFF group and twice in the DCIA group (*p* = 0.401). Dehiscence was registered 13 times in the plate group, eleven times in the FFF group and never in the DCIA group within 100 days (*p* = 0.121). Within the late interval, dehiscence occurred three times in the plate group, three times in the FFF group and once in the DCIA group (*p* = 0.955). Intraoral bone exposure occurred once each in the plate group and four times in the FFF group within 100 days (*p* = 0.818) and four times in the plate group and four times in the FFF group in the late interval (*p* = 0.854). Cutaneous perforation within 100 days was only seen in the plate group six times (*p* = 0.004). In the late interval, it was registered seven in the plate group, five times in the FFF group and once in the DCIA group (*p* = 0.617). Screw loosening was only seen once in the DCIA group within 100 days (*p* = 0.004). In the late interval, it became evident each four times in the plate and FFF groups and once in the DCIA group (*p* = 0.972). Plate fracture was only seen in the FFF group three times within 100 postoperative days (*p* = 0.172). In the late interval, it was registered twice in the plate group, four times in the FFF group and never in the DCIA group (*p* = 0.371).

Detailed results for the incidences of postoperative fistula, dehiscence and cutaneous perforation formation within the subgroups with regard to all analysed possible confounding factors are shown in the Supplementary Tables S1–3.

The incidences of postoperative fistula, dehiscence and cutaneous perforation within the subgroups with regard to the most relevant possible confounding factors on the basis of the Supplementary Tables S1–3 are summarized in Table 3 (age, BMI and history of radiation represented as “TotalGy”). Overall, history of radiation with the total amount of gray (Gy) was significantly associated with all complications. Within the differentiation of the subgroups, the mandibular reconstruction technique with intraoperatively bend reconstruction plates showed a strong association between the history of radiation and the incidence of fistula formation.

**Table 3:** Mean and standard deviation (SD) of postoperative fistula, dehiscence and cutaneous perforation

			Postoperative fistula					Postoperative dehiscence					Postoperative cutaneous perforation				
			No		Yes			No		Yes			No		Yes		
			Mean	± SD	Mean	± SD	*p* value	Mean	± SD	Mean	±SD	*p* value	Mean	± SD	Mean	± SD	*p* value
Reconstruction technique	Rec. plate intraop. bend	Age	63	12	68	10	0.408	64	12	59	10	0.363	65	12	59	11	0.247
		BMI	22.7	5.5	23.6	3.4	0.733	23.8	5.0	18.1	2.9	0.006	23.3	5.5	22.3	4.0	0.615
		TotalGy	46.1	30.8	89.5	46.3	0.058	51.8	37.8	60.8	34.0	0.664	49.0	39.5	63.7	29.4	0.255
	Rec. plate pre-bend	Age	65	15	68	8	0.676	65	15	66	9	0.764	65	14	65	10	0.712
		BMI	25.1	5.7	21.8	2.5	0.110	24.2	5.5	25.6	5.2	0.536	24.4	5.4	25.0	5.8	1.000
		TotalGy	36.8	34.8	45.3	31.5	0.326	35.2	35.4	44.5	31.0	0.245	38.9	34.1	32.7	37.7	1.000
	FFF conv.	Age	60	11	57	12	0.339	60	11	56	11	0.174	59	11	56	4	0.253
		BM*I*	23.1	5.1	22.1	2.5	0.528	22.7	4.8	23.5	3.8	0.356	23.2	4.6	18.2	1.5	0.004
		TotalGy	45.9	32.0	57.1	28.5	0.554	46.8	30.4	57.9	35.3	0.244	47.6	32.1	65.1	4.3	0.118
	FFF CAD/CAM	Age	53	14	55	1	0.886	53	14	50	14	0.490	52	13	65		
		BMI	22.8	2.7	27.6	10.0	0.745	23.0	3.7	23.9	2.8	0.490	23.0	3.6	25.3		
		TotalGy	42.9	31.3	66.2	3.1	0.345	44.8	30.7	43.8	38.0	1.000	43.9	31.1	64.0		
	DCIA	Age	50	16	56	12	0.627	51	15	52			51	15	52		
		BMI	26.1	5.1	28.0	3.6	0.477	26.6	4.8	22.1			26.6	4.8	22.1		
		TotalGy	6.7	20.6	16.0	32.0	0.682	5.8	19.3	64.0			5.8	19.3	64.0		
	Total	Age	59	14	60	12	0.630	59	14	59	11	0.399	59	14	60	9	0.589
		BMI	23.8	5.1	23.3	3.8	0.503	23.8	5.0	23.2	4.7	0.504	23.9	4.9	22.2	4.4	0.082
		TotalGy	38.4	33.3	56.1	36.8	0.043	39.3	34.6	53.0	32.4	0.043	39.7	34.8	57.5	28.2	0.018

*Rec.* reconstruction, *intraop.* intraoperatively, *FFF* free fibula flap, *conv.* conventional, *DCIA flap* deep circumflex iliac artery, *CAD/CAM* computer-aided design/computer-aided manufacturing, *BMI* body mass index, *Gy* gray

A low BMI significantly more often led to postoperative dehiscence when intraoperatively bend reconstruction plates were used (18.1 ± 2.5 kg/m^2^; *p* = 0.006). A low BMI was also associated with cutaneous perforation within the CAD/CAM FFF subgroup (18.2 ± 1.5 kg/m^2^; *p* = 0.004).

Distinguishing between the timing of radiation therapy, a positive history of radiation therapy was significantly associated with wound healing disturbances like postoperative fistula or dehiscence formation (*p* = 0.009, Table S1, and *p* = 0.027, Table S2). Cutaneous perforation occurred more frequently in cases with intraoperatively bend reconstruction plates and conventional FFF reconstructions compared with pre-bend plates and CAD/CAM FFF in the Fisher exact tests for difference in frequency of this complication between reconstruction type within the subgroups (*p* = 0.055, Table S3) in cases with a positive history of radiation therapy.

## Discussion

This retrospective study investigated the most common postoperative complications after mandibulectomy and simultaneous reconstruction using only reconstruction plates, free fibula flaps or DCIA flaps in 190 cases. Potential risk factors for the occurrence of postoperative complications were analysed.

### Frequency and nature of complications observed

The overall incidence of complications after mandibular reconstruction varies between 8 and 57% in the literature [11, 17–19]. Lee et al. described fistula formation (8.4%), hardware plate exposure (7.1%) and flap wound infections (6.5%) to be the most common complications. Our complication rates are higher for all mentioned complications, except for flap wound infection. This might be attributed to somewhat different definitions of complications or a differing complexity of the studied cohorts. As stated earlier, our understanding and precise definition of fistula and dehiscence might be different to the definitions authors have used to describe their wound-healing disorders [18, 20, 21]. Infection of the flap (wound) is rarely seen in daily routine at our department, which might be attributed to an established protocol for the intra- and postoperative administration of intravenous antibiotic therapy and the common standards of hygiene [22].

Plate fracture after mandibular reconstruction has been reported in the literature. Rendenbach et al. analysed the stability and strength of miniplates, common reconstruction plates and CAD/CAM plates in an in vitro study. They demonstrated no significant difference up to a load of 300 N [23]. There exists a diversity of results regarding stability, associated complications and costs of used fixation plates for the FFF and DCIA flap [24–30]. The bottom line of most manuscripts on this topic is that miniplate osteosynthesis remains the fixation system of choice for the FFF due to a low rate of associated complications and its good clinical handling. Cost-effective analyses and long-term results with an adequate scientific quality of patient-specific, laser-sintered titanium plates need to be conducted and are expected in the future [31].

### Impact of the choice of reconstructive technique on the occurrence of complications

Markiewicz et al. described an overall high success rate of 94.8% of free flap reconstruction for the mandible using the FFF, the DCIA flap, the osteocutaneous radial forearm flap and the scapula flap. No significant difference in failure was found comparing FFF and DCIA in their meta-analysis encompassing 17 studies [1].

On the other hand, Lodders et al. described, for example, a higher complication rate in FFF compared with other free flaps [18]. This is in concordance with our results, where we registered more fistula and dehiscence formation in comparison with the FFF group.

Further, the extent of mandibular reconstruction was also identified as a relative risk factor in a literature review by Sadr-Eshkevari and colleagues. In their study, crossing the midline seemed to favour plate exposure, a phenomenon that has also been described by others [32–34]. Plate exposure is a severe complication that necessitates a critical review of the treatment plan, and a new operation including plate removal and secondary reconstruction [35]. Wei et al. reported a plate exposure rate of 46.15% in a retrospective analysis of patients who had been reconstructed with a reconstruction plate and soft tissue free flap [36]. Their high incidence of plate exposure was probably due to patient selection, including only patients with recurrent carcinomas and stage IV tumours. In the authors’ experience, in cases of mandibular reconstruction (Fig. 1) using the reconstruction plate, the re-fixation of the temporarily released supramyohyoidal muscles is important from the perspective of soft tissue in addition to functional considerations. Further, the plate design with a suggested retrognathic position and a counterbored plate position on the remaining mandible might release some of the soft tissue tension over the reconstruction plate and reduce the dead space below the plate [33]. Pre-bending the plate reduces operation time and increases the accuracy of the reconstructive result, as shown by others [37, 38]. Despite following all these known and described suggestions, both plate groups (pre-bend plate and intraoperatively bend plate) had high rates of complications, with the lower incidence in the pre-bend plate subgroup.

### Impact of total radiation dosage on the occurrence of complications

The highest overall recipient complication rate, though, was observed after the use of reconstruction plates. However, this observation might also be attributable to selection bias in this group, as seen in the study by Wei et al. [36]. The plate group had the highest number of malignancies and, if required in accordance with the current guidelines [39], consecutive adjuvant radiation therapy. Our FFF group had the highest number of cases of osteoradionecrosis (ORN). Both underlying indication and clinical situation favour wound-healing disturbances in the head and neck area as reported in the literature [40, 41]. In our resent study, postoperative fistula formation and development of dehiscence were more frequently seen in patients with a positive history of radiation therapy (*p* = 0.009 and *p* = 0.027, respectively), rather than in the study population with adjuvant radiation therapy (*p* = 0.441 and *p* = 0.670, respectively) (Tables S1 and 2). On the other hand, cutaneous perforation was not significantly influenced with regard to the timing of radiation therapy and reconstruction technique (Table S3). In this context, a positive history of radiation therapy is a known risk factor for complications [42, 43] that has led us to adapt the concept of double free flap reconstructions [12, 44]. In a recent systematic review and meta-analysis, Mijiti et al. demonstrated an odds ratio of 1.90 that suggests a significantly increased rate for fistula formation [45]. On the other hand, the development of wound dehiscence or plate exposure was not significantly associated with a positive history of radiation. But only a minority of included studies have addressed these complications.

Overall, these unfavourable clinical situations and above-mentioned complications can be caused by secondary tissue and vascular fibrosis, altered extracellular matrix remodelling and changes in vasculature and local host defence peptides [46–48]. In accordance with this assumption, Lonie et al. described a higher recipient site complication rate for the DCIA flap compared with the FFF in a comparative literature review [49]. In their included studies, the incidence of radiotherapy was higher in the DCIA group, which is the opposite of our cohort, where the DCIA flap was almost exclusively applied for mandibular reconstructions of benign processes (Table 1).

This might lead to a key feature that has to be considered when it comes to reconstruction in irradiated head and neck areas: tensionless wound closure with robust free flap selection. An estimation of potential risk factors that favour the incidence of complications, such as fistula/ dehiscence formation or cutaneous perforation, must be addressed in a thorough preoperative, clinical examination of the patient.

### Patient constitution and behaviour-related risk factors

The analysis of risk factors that contribute to the incidence of postoperative complications is difficult. Most studies analysed a heterogeneous cohort with a varying number of cases and microvascular free flaps. Overall, several risk factors have already been assumed for microvascular free flap transfer, including age, tobacco use, comorbidity, previous flap loss, ASA status and operating time [50–53].

In this study, low BMI and total amount of radiation dosage (Gy) emerged to be the most relevant risk factors (Table 3). Lo et al. also described an increased recipient site complication rate in patients with low BMI [19]. This is probably due to a decreased amount of subcutaneous fat tissue and muscle layers that could buffer the permanent mechanical stress during mandibular movements. An increased infection rate in obese patients with high BMI could not be described by Khan et al. [54].

ASA status showed a significant influence only on fistula formation (*p* = 0.010; Supplementary Table S1). As described previously and by others, ASA status is also a risk factor for flap loss [53]. However, this finding must be interpreted with caution, since this parameter reflects the constitution of the patients and therefore is biased by other underlying diseases that might also contribute to flap loss or complications.

Smoking and diabetes mellitus had no significant influence on fistula or dehiscence formation (Supplementary Tables S1–3). Smoking, however, had a strong trend towards the development of postoperative cutaneous perforation (Supplementary table S3). Within the limits of this study, one could again argue that a potential selection bias has contributed to the coincidence of cutaneous perforation in the plate and FFF reconstruction subgroups, as smoking remains one of the main risk factors for the development of oral squamous cell carcinoma (OSCC).

### Limitations

The presented study is a retrospective study with limitations that go along with this kind of study type. The provided clinical data has a potential for variability in reports. The authors attempted to minimize bias through definitions and data collection by a single researcher (DH). Secondly, the enrolled patients might not be representative of the entire population, although the study cohort reflects the regular distribution as reported in the literature. Thirdly, this study focused on the most common complications in the head and neck area after mandibular reconstruction and excluded donor site comorbidity, which might be associated with flap harvesting, as reported by others.

### Treatment algorithm

**Fig. 2:**
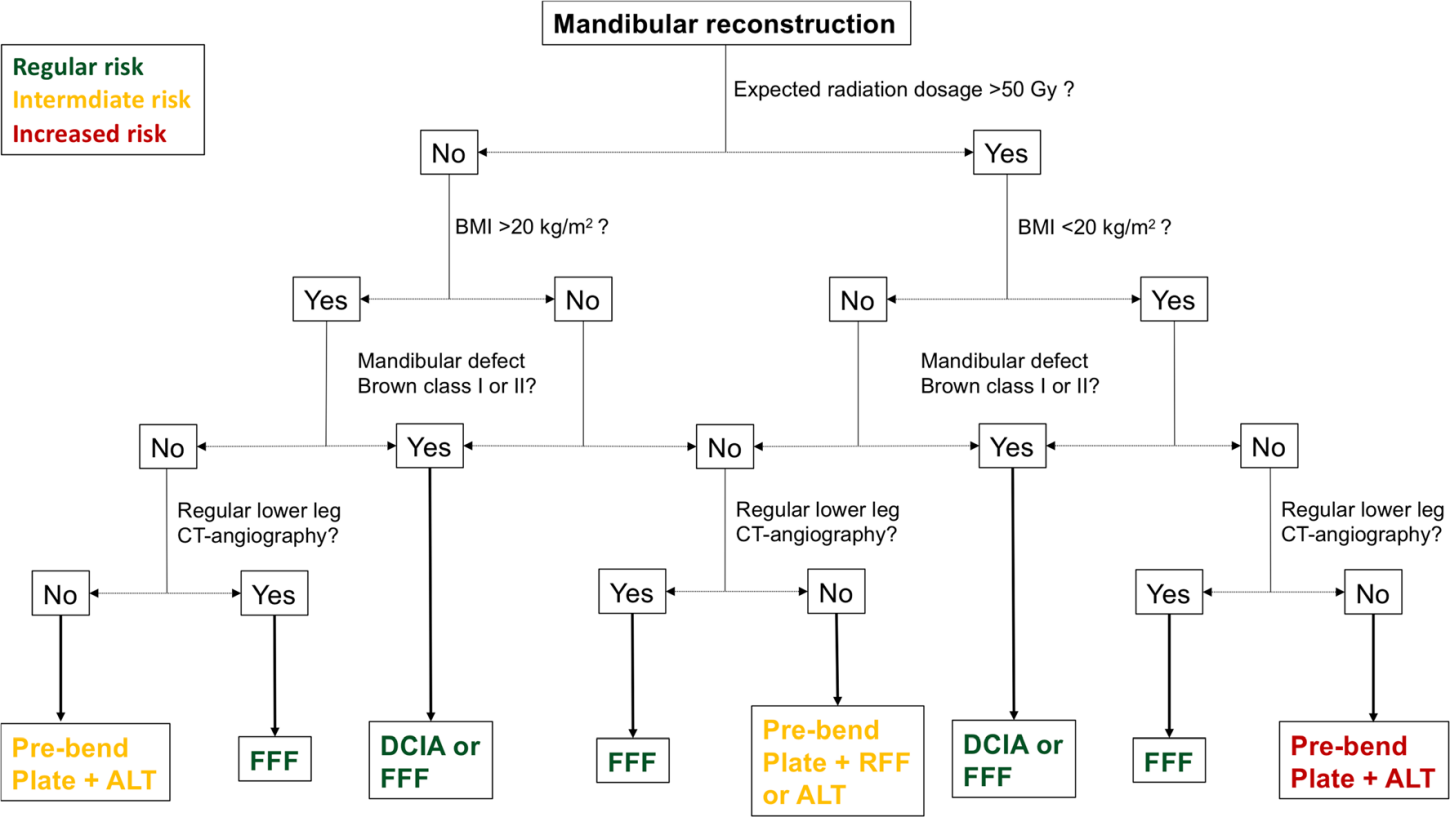
Treatment algorithm for mandibular reconstructions on the basis of our results. Brown’s classification was used to describe the mandibular defect situation [55]

Despite their different complication profiles, all techniques used in this study have a scope of application, and the choice of reconstruction method should be made for each individual patient. Within the limitations of this retrospective study and the experience of the authors, the different complication profiles and potential risk factors have helped us develop a treatment algorithm that can help in the decision-making process, which is illustrated in Fig. 2. This algorithm incorporates the most relevant risk factors (radiation dosage and patient BMI) and the expected mandibular defect as defined by Brown et al. [55].

## Conclusions

Mandibular reconstruction is a safe procedure within the limits of underlying indication and applied reconstructive technique. Age, low BMI and total amount of radiation dosage (Gy) seem to have a significant impact on postoperative fistula, dehiscence and cutaneous perforation formation. These risk factors should be kept in mind in reconstructive technique selection. CAD/CAM FFF and preoperatively bend reconstruction plates had fewer complications than the corresponding conventional reconstructive technique. Free bone transplants offer the best functional and anatomical reconstruction option and have a lower risk of recipient complications than reconstruction plates. This reconstructive technique should therefore be applied whenever possible.

## Electronic supplementary material

ESM 1 (DOCX 59 kb)

## Abbreviations

ASA: American Society of Anaesthesiologists status
BMI: body mass index
CAD/CAM: computer-aided design and computer-aided manufacturing
CT: computed tomography
DCIA: free iliac crest flap
FFF: free fibula flap
Gy: gray
MRONJ: medication-related necrosis of the jaw
OSCC: oral squamous cell carcinoma
ORN: osteoradionecrosis
