# Supplementary material for: Retrospective analysis of complications in 190 mandibular resections and simultaneous reconstructions with free fibula flap, iliac crest flap or reconstruction plate: a comparative single centre study
Source: Clin Oral Investig. 2020 Oct 6;25(5):2905–14. doi: 10.1007/s00784-020-03607-8 (PMC8060197; doi:10.1007/s00784-020-03607-8)
Supplement: Supplementary file 1 — (DOCX 59 kb) [file 784_2020_3607_MOESM1_ESM.docx]

**List of Supplementary tables**

**Journal:** Journal of Clinical Oral Investigations

**“Retrospective analysis of complications in 190 mandibular resections and simultaneous reconstructions with free fibula flap, iliac crest flap or reconstruction plate: a comparative single centre study”**

Lucas M. Ritschl ^1^, MD DDS; Thomas Mücke ^2^, MD DDS; Diandra Hart ^1^, DMD; Tobias Unterhuber^1^, MD DMD; Victoria Kehl ^3^, PhD; Klaus-Dietrich Wolff ^1^, MD DDS; Andreas M. Fichter ^1^, MD DDS

**Corresponding author:**

Lucas M. Ritschl, MD DDS

Department of Oral and Maxillofacial Surgery

Klinikum rechts der Isar, Technical University of Munich

Ismaninger Straße 22, 81675 Munich, Germany

E-mail: [lucas.ritschl@tum.de](mailto:lucas.ritschl@tum.de)

| **Supplementary table S1.** Frequency of postoperative fistula within the subgroups and analysis of potential risk factors. | | | | | | | | | | | | | | | |
| --- | --- | --- | --- | --- | --- | --- | --- | --- | --- | --- | --- | --- | --- | --- | --- |
|  | | **Reconstruction technique** | | | | | | | | | | | |  |  |
|  |  | **Rec. plate intraop. bend** | | **Rec. plate pre-bend** | | **FFF conventional** | | **FFF CAD/CAM** | | **DCIA** | | **Total** | | **p-value** | |
|  |  | N | % | N | % | N | % | N | % | N | % | N | % | & | § |
| **Total** | *no post-OP fistula* | 31 | 83.8 | 33 | 82.5 | 47 | 74.6 | 24 | 92.3 | 20 | 83.3 | 155 | 81.6 |  | 0.441 # |
|  | *post-OP fistula* | 6 | 16.2 | 7 | 17.5 | 16 | 25.4 | 2 | 7.7 | 4 | 16.7 | 35 | 18.4 |  |  |
| **Gender** | *female* | 2 | 12.5 | 2 | 13.3 | 3 | 13.6 | 0 | 0.0 | 1 | 9.1 | 8 | 11.1 | 0.939 | 0.042 * |
|  | *male* | 4 | 19.0 | 5 | 20.0 | 13 | 31.7 | 2 | 11.1 | 3 | 23.1 | 27 | 22.9 | 0.523 |  |
| **ASA-status** | *I* | 0 | 0.0 | 0 | 0.0 | 0 | 0.0 | 0 | 0.0 | 0 | 0.0 | 0 | 0.0 |  | 0.010 |
|  | *II* | 3 | 15.0 | 2 | 9.5 | 11 | 27.5 | 2 | 12.5 | 4 | 33.3 | 22 | 20.2 | 0.314 |  |
|  | *III* | 3 | 21.4 | 5 | 31.3 | 4 | 23.5 | 0 | 0.0 | 0 | 0.0 | 12 | 21.1 | 0.573 |  |
|  | *IV* | 0 | 0.0 | 0 | 0.0 | 1 | 100.0 | 0 | 0.0 | 0 | 0.0 | 1 | 100.0 |  |  |
| **Smoker** | *no* | 4 | 26.7 | 4 | 19.0 | 8 | 24.2 | 1 | 7.7 | 1 | 6.7 | 18 | 18.6 | 0.472 | 0.961 * |
|  | *yes* | 2 | 9.1 | 3 | 15.8 | 8 | 26.7 | 1 | 7.7 | 3 | 33.3 | 17 | 18.3 | 0.298 |  |
| **Diabetes Mellitus** | *no* | 5 | 15.2 | 6 | 18.2 | 15 | 25.0 | 1 | 4.3 | 4 | 19.0 | 31 | 18.2 | 0.272 | 0.750 |
|  | *yes* | 1 | 25.0 | 1 | 16.7 | 1 | 33.3 | 1 | 50.0 | 0 | 0.0 | 4 | 22.2 | 0.812 |  |
| **Entity‎/ Indication** | *OSCC* | 4 | 14.3 | 3 | 11.5 | 8 | 30.8 | 1 | 10.0 | 0 | 0.0 | 16 | 17.4 | 0.389 | 0.198 |
|  | *MRONJ* | 0 | 0.0 | 1 | 50.0 | 3 | 75.0 | 0 | 0.0 | 0 | 0.0 | 4 | 44.4 | 0.492 |  |
|  | *ORN* | 2 | 33.3 | 2 | 50.0 | 5 | 20.0 | 1 | 12.5 | 2 | 66.7 | 12 | 26.1 | 0.229 |  |
|  | *Keratocyst* | 0 | 0.0 | 0 | 0.0 | 0 | 0.0 | 0 | 0.0 | 2 | 20.0 | 2 | 16.7 | 1.000 |  |
|  | *Ameloblastoma* | 0 | 0.0 | 0 | 0.0 | 0 | 0.0 | 0 | 0.0 | 0 | 0.0 | 0 | 0.0 |  |  |
|  | *Aneurysmatic bone cyst* | 0 | 0.0 | 0 | 0.0 | 0 | 0.0 | 0 | 0.0 | 0 | 0.0 | 0 | 0.0 |  |  |
|  | *Osteosarcoma* | 0 | 0.0 | 0 | 0.0 | 0 | 0.0 | 0 | 0.0 | 0 | 0.0 | 0 | 0.0 |  |  |
|  | *OM* | 0 | 0.0 | 0 | 0.0 | 0 | 0.0 | 0 | 0.0 | 0 | 0.0 | 0 | 0.0 |  |  |
|  | *others* | 0 | 0.0 | 1 | 33.3 | 0 | 0.0 | 0 | 0.0 | 0 | 0.0 | 1 | 14.3 | 0.571 |  |
| **Malignancy** | *no* | 2 | 25.0 | 3 | 27.3 | 8 | 22.2 | 1 | 6.3 | 4 | 18.2 | 18 | 19.4 | 0.606 | 0.745 * |
|  | *yes* | 4 | 13.8 | 4 | 13.8 | 8 | 29.6 | 1 | 10.0 | 0 | 0.0 | 17 | 17.5 | 0.484 |  |
| **History of radiation** | *no* | 3 | 12.5 | 3 | 9.7 | 4 | 17.4 | 1 | 7.7 | 3 | 13.6 | 14 | 12.4 | 0.919 | 0.009 * |
|  | *yes* | 3 | 23.1 | 4 | 44.4 | 12 | 30.0 | 1 | 7.7 | 1 | 50.0 | 21 | 27.3 | 0.270 |  |
| **Adjuvant chemo-therapy** | *no* | 5 | 17.2 | 7 | 21.2 | 15 | 25.9 | 2 | 9.1 | 4 | 18.2 | 33 | 20.1 | 0.586 | 0.175 |
|  | *yes* | 1 | 12.5 | 0 | 0.0 | 1 | 20.0 | 0 | 0.0 | 0 | 0.0 | 2 | 7.7 | 0.828 |  |
| **Adjuvant radiation** | *no* | 0 | 0.0 | 6 | 26.1 | 12 | 24.0 | 1 | 4.8 | 4 | 18.2 | 23 | 17.0 | 0.036 | 0.441 * |
|  | *yes* | 6 | 33.3 | 1 | 5.9 | 4 | 30.8 | 1 | 20.0 | 0 | 0.0 | 12 | 21.8 | 0.235 |  |
| \| **Abbreviations:** Rec. = reconstruction; FFF = free fibula flap; DCIA = deep circumflex iliac artery flap; CAD/CAM = computer-aided design/computer-aided manufacturing; intraop. = intraoperatively; OSCC = oral squamous cell carcinoma; MRONJ = medication related necrosis of the jaw; ORN = osteoradionecrosis; OM = osteomyelitis  & Fisher exact test for difference in frequency of postoperative fistula between reconstruction type within subgroups \| \| --- \| \| § Fisher exact test for difference in frequency of postoperative fistula between subgroups over all reconstruction techniques, unless otherwise stated \| \| # Fisher exact test for difference in frequency of postoperative fistula over all reconstruction techniques \| \| * Chi square test for difference in frequency of postoperative fistula between subgroups over all reconstruction techniques \| | | | | | | | | | | | | | | | |

| **Supplementary table S2.** Frequency of postoperative dehiscence within the subgroups and analysis of potential risk factors. | | | | | | | | | | | | | | | |
| --- | --- | --- | --- | --- | --- | --- | --- | --- | --- | --- | --- | --- | --- | --- | --- |
|  | | **Reconstruction technique** | | | | | | | | | | | |  |  |
|  |  | **Rec. plate intraop. bend** | | **Rec. plate pre-bend** | | **FFF conventional** | | **FFF CAD/CAM** | | **DCIA** | | **Total** | | **p-value** | |
|  |  | N | % | N | % | N | % | N | % | N | % | N | % | & | § |
| **Total** | *no post-OP dehiscence* | 31 | 83.8 | 29 | 74.4 | 52 | 82.5 | 23 | 88.5 | 23 | 95.8 | 158 | 83.6 |  | 0.246 # |
|  | *post-OP dehiscence* | 6 | 16.2 | 10 | 25.6 | 11 | 17.5 | 3 | 11.5 | 1 | 4.2 | 31 | 16.4 |  |  |
| **Gender** | *female* | 4 | 25.0 | 5 | 33.3 | 6 | 27.3 | 1 | 12.5 | 1 | 9.1 | 17 | 23.6 | 0.645 | 0.036 * |
|  | *male* | 2 | 9.5 | 5 | 20.8 | 5 | 12.2 | 2 | 11.1 | 0 | 0.0 | 14 | 12.0 | 0.522 |  |
| **ASA-status** | *I* | 1 | 33.3 | 0 | 0.0 | 0 | 0.0 | 1 | 20.0 | 0 | 0.0 | 2 | 8,7% | 0.375 | 0.695 |
|  | *II* | 3 | 15.0 | 5 | 23.8 | 9 | 22.5 | 1 | 6.3 | 1 | 8.3 | 19 | 17.4 | 0.549 |  |
|  | *III* | 2 | 14.3 | 5 | 33.3 | 2 | 11.8 | 1 | 20.0 | 0 | 0.0 | 10 | 17.9 | 0.468 |  |
|  | *IV* | 0 | 0.0 | 0 | 0.0 | 0 | 0.0 | 0 | 0.0 | 0 | 0.0 | 0 | 0.0 |  |  |
| **Smoker** | *no* | 1 | 6.7 | 4 | 20.0 | 6 | 18.2 | 2 | 15.4 | 1 | 6.7 | 14 | 14.6 | 0.727 | 0.493 * |
|  | *yes* | 5 | 22.7 | 6 | 31.6 | 5 | 16.7 | 1 | 7.7 | 0 | 0.0 | 17 | 18.3 | 0.278 |  |
| **Diabetes Mellitus** | *no* | 6 | 18.2 | 10 | 30.3 | 11 | 18.3 | 3 | 13.0 | 1 | 4.8 | 31 | 18.2 | 0.201 | 0.080 |
|  | *yes* | 0 | 0.0 | 0 | 0.0 | 0 | 0.0 | 0 | 0.0 | 0 | 0.0 | 0 | 0.0 |  |  |
| **Entity‎/ Indication** | *OSCC* | 4 | 14.3 | 6 | 23.1 | 2 | 7.7 | 0 | 0.0 | 1 | 50.0 | 13 | 14.1 | 0.155 | 0.223 |
|  | *MRONJ* | 0 | 0.0 | 0 | 0.0 | 1 | 25.0 | 0 | 0.0 | 0 | 0.0 | 1 | 11.1 | 1.000 |  |
|  | *ORN* | 2 | 33.3 | 2 | 50.0 | 8 | 32.0 | 2 | 25.0 | 0 | 0.0 | 14 | 30.4 | 0.814 |  |
|  | *Keratocyst* | 0 | 0.0 | 0 | 0.0 | 0 | 0.0 | 0 | 0.0 | 0 | 0.0 | 0 | 0.0 |  |  |
|  | *Ameloblastoma* | 0 | 0.0 | 1 | 50.0 | 0 | 0.0 | 1 | 100.0 | 0 | 0.0 | 2 | 18.2 | 0.109 |  |
|  | *Aneurysmatic bone cyst* | 0 | 0.0 | 0 | 0.0 | 0 | 0.0 | 0 | 0.0 | 0 | 0.0 | 0 | 0.0 |  |  |
|  | *Osteosarcoma* | 0 | 0.0 | 0 | 0.0 | 0 | 0.0 | 0 | 0.0 | 0 | 0.0 | 0 | 0.0 |  |  |
|  | *OM* | 0 | 0.0 | 1 | 50.0 | 0 | 0.0 | 0 | 0.0 | 0 | 0.0 | 1 | 11.1 | 0.222 |  |
|  | *others* | 0 | 0.0 | 0 | 0.0 | 0 | 0.0 | 0 | 0.0 | 0 | 0.0 | 0 | 0.0 |  |  |
| **Malignancy** | *no* | 2 | 25.0 | 4 | 36.4 | 9 | 25.0 | 3 | 18.8 | 0 | 0.0 | 18 | 19.4 | 0.027 | 0.281 * |
|  | *yes* | 4 | 13.8 | 6 | 21.4 | 2 | 7.4 | 0 | 0.0 | 1 | 50.0 | 13 | 13.5 | 0.183 |  |
| **History of radiation** | *no* | 2 | 8.3 | 8 | 25.8 | 1 | 4.3 | 1 | 7.7 | 1 | 4.5 | 13 | 11.5 | 0.111 | 0.027 * |
|  | *yes* | 4 | 30.8 | 2 | 25.0 | 10 | 25.0 | 2 | 15.4 | 0 | 0.0 | 18 | 23.7 | 0.902 |  |
| **Adjuvant chemo-therapy** | *no* | 6 | 20.7 | 8 | 25.0 | 9 | 15.5 | 3 | 13.6 | 0 | 0.0 | 26 | 16.0 | 0.100 | 0.775 |
|  | *yes* | 0 | 0.0 | 2 | 28.6 | 2 | 40.0 | 0 | 0.0 | 1 | 50.0 | 5 | 19.2 | 0.146 |  |
| **Adjuvant radiation** | *no* | 4 | 21.1 | 5 | 22.7 | 9 | 18.0 | 3 | 14.3 | 0 | 0.0 | 21 | 15.7 | 0.146 | 0.670 |
|  | *yes* | 2 | 11.1 | 5 | 29.4 | 2 | 15.4 | 0 | 0.0 | 1 | 50.0 | 10 | 18.2 | 0.318 |  |
| \| **Abbreviations:** Rec. = reconstruction; FFF = free fibula flap; DCIA flap = deep circumflex iliac artery; CAD/CAM = computer-aided design/computer-aided manufacturing; intraop. = intraoperatively; OSCC = oral squamous cell carcinoma; MRONJ = medication related necrosis of the jaw; ORN = osteoradionecrosis; OM = osteomyelitis  & Fisher exact test for difference in frequency of postoperative dehiscence between reconstruction type within subgroups \| \| --- \| \| § Fisher exact test for difference in frequency of postoperative dehiscence between subgroups over all reconstruction techniques, unless otherwise stated \| \| # Fisher exact test for difference in frequency of postoperative dehiscence over all reconstruction techniques \| \| * Chi square test for difference in frequency of postoperative dehiscence between subgroups over all reconstruction techniques \| | | | | | | | | | | | | | | | |

| **Supplementary table S3.** Frequency of postoperative cutaneous perforation within the subgroups and analysis of potential risk factors. | | | | | | | | | | | | | | | |
| --- | --- | --- | --- | --- | --- | --- | --- | --- | --- | --- | --- | --- | --- | --- | --- |
|  | | **Reconstruction technique** | | | | | | | | | | | |  |  |
|  |  | **Rec. plate intraop. bend** | | **Rec. plate pre-bend** | | **FFF conventional** | | **FFF CAD/CAM** | | **DCIA** | | **Total** | | **p-value** | |
|  |  | N | % | N | % | N | % | N | % | N | % | N | % | & | § |
| **Total** | *no cutaneous perforation* | 27 | 75.0 | 36 | 90.0 | 59 | 93.7 | 25 | 96.2 | 23 | 95.8 | 170 | 89.9 |  | 0.039 # |
|  | *cutaneous perforation* | 9 | 25.0 | 4 | 10.0 | 4 | 6.3 | 1 | 3.8 | 1 | 4.2 | 19 | 10.1 |  |  |
| **Gender** | *female* | 5 | 31.3 | 1 | 6.7 | 0 | 0.0 | 0 | 0.0 | 1 | 9.1 | 7 | 9.7 | 0.018 | 0.906 * |
|  | *male* | 4 | 20.0 | 3 | 12.0 | 4 | 9.8 | 1 | 5.6 | 0 | 0.0 | 12 | 10.3 | 0.470 |  |
| **ASA-status** | *I* | 1 | 50.0 | 0 | 0.0 | 0 | 0.0 | 0 | 0.0 | 0 | 0.0 | 1 | 4.5 | 0.091 | 0.112 |
|  | *II* | 5 | 25.0 | 0 | 0.0 | 3 | 7.5 | 1 | 6.3 | 1 | 8.3 | 10 | 9.2 | 0.084 |  |
|  | *III* | 3 | 21.4 | 4 | 25.0 | 0 | 0.0 | 0 | 0.0 | 0 | 0.0 | 7 | 12.3 | 0.130 |  |
|  | *IV* | 0 | 0.0 | 0 | 0.0 | 1 | 100.0 | 0 | 0.0 | 0 | 0.0 | 1 | 100.0 |  |  |
| **Smoker** | *no* | 2 | 13.3 | 2 | 9.5 | 0 | 0.0 | 1 | 7.7 | 1 | 6.7 | 6 | 6.2 | 0.198 | 0.069 * |
|  | *yes* | 7 | 33.3 | 2 | 10.5 | 4 | 13.3 | 0 | 0.0 | 0 | 0.0 | 13 | 14.1 | 0.055 |  |
| **Diabetes Mellitus** | *no* | 8 | 25.0 | 4 | 12.1 | 4 | 6.7 | 1 | 4.3 | 1 | 4.8 | 18 | 10.7 | 0.075 | 0.700 |
|  | *yes* | 1 | 25.0 | 0 | 0.0 | 0 | 0.0 | 0 | 0.0 | 0 | 0.0 | 1 | 5.6 | 0.667 |  |
| **Entity‎/ Indication** | *OSCC* | 6 | 22.2 | 3 | 11.5 | 4 | 15.4 | 0 | 0.0 | 1 | 50.0 | 14 | 15.4 | 0.276 | 0.646 |
|  | *MRONJ* | 0 | 0.0 | 0 | 0.0 | 0 | 0.0 | 0 | 0.0 | 0 | 0.0 | 0 | 0.0 |  |  |
|  | *ORN* | 3 | 50.0 | 0 | 0.0 | 0 | 0.0 | 1 | 12.5 | 0 | 0.0 | 4 | 8.7 | 0.009 |  |
|  | *Keratocyst* | 0 | 0.0 | 0 | 0.0 | 0 | 0.0 | 0 | 0.0 | 0 | 0.0 | 0 | 0.0 |  |  |
|  | *Ameloblastoma* | 0 | 0.0 | 0 | 0.0 | 0 | 0.0 | 0 | 0.0 | 0 | 0.0 | 0 | 0.0 |  |  |
|  | *Aneurysmatic bone cyst* | 0 | 0.0 | 0 | 0.0 | 0 | 0.0 | 0 | 0.0 | 0 | 0.0 | 0 | 0.0 |  |  |
|  | *Osteosarcoma* | 0 | 0.0 | 0 | 0.0 | 0 | 0.0 | 0 | 0.0 | 0 | 0.0 | 0 | 0.0 |  |  |
|  | *OM* | 0 | 0.0 | 1 | 50.0 | 0 | 0.0 | 0 | 0.0 | 0 | 0.0 | 1 | 11.1 | 0.444 |  |
|  | *others* | 0 | 0.0 | 0 | 0.0 | 0 | 0.0 | 0 | 0.0 | 0 | 0.0 | 0 | 0.0 |  |  |
| **Malignancy** | *no* | 3 | 37.5 | 1 | 9.1 | 0 | 0.0 | 1 | 6.3 | 0 | 0.0 | 5 | 5.4 | 0.001 | 0.035 * |
|  | *yes* | 6 | 21.4 | 3 | 10.3 | 4 | 14.8 | 0 | 0.0 | 1 | 50.0 | 14 | 14.6 | 0.255 |  |
| **History of radiation** | *no* | 4 | 16.7 | 4 | 12.9 | 0 | 0.0 | 0 | 0.0 | 1 | 4.5 | 9 | 8.0 | 0.157 | 0.244 * |
|  | *yes* | 5 | 41.7 | 0 | 0.0 | 4 | 10.0 | 1 | 7.7 | 0 | 0.0 | 10 | 13.2 | 0.055 |  |
| **Adjuvant chemo-therapy** | *no* | 8 | 28.6 | 4 | 12.1 | 4 | 6.9 | 1 | 4.5 | 0 | 0.0 | 17 | 10.4 | 0.012 | 1.000 |
|  | *yes* | 1 | 12.5 | 0 | 0.0 | 0 | 0.0 | 0 | 0.0 | 1 | 50.0 | 2 | 7.7 | 0.200 |  |
| **Adjuvant radiation** | *no* | 4 | 22.2 | 2 | 8.7 | 4 | 8.0 | 1 | 4.8 | 0 | 0.0 | 11 | 8.2 | 0.165 | 0.188 * |
|  | *yes* | 5 | 27.8 | 2 | 11.8 | 0 | 0.0 | 0 | 0.0 | 1 | 50.0 | 8 | 14.5 | 0.100 |  |
| \| **Abbreviations:** Rec. = reconstruction; FFF = free fibula flap; DCIA flap = deep circumflex iliac artery; CAD/CAM = computer-aided design/computer-aided manufacturing; intraop. = intraoperatively; OSCC = oral squamous cell carcinoma; MRONJ = medication related necrosis of the jaw; ORN = osteoradionecrosis; OM = osteomyelitis  & Fisher exact test for difference in frequency of postoperative cutaneous perforation between reconstruction type within subgroups \| \| --- \| \| § Fisher exact test for difference in frequency of postoperative cutaneous perforation between subgroups over all reconstruction techniques, unless otherwise stated \| \| # Fisher exact test for difference in frequency of postoperative cutaneous perforation over all reconstruction techniques \| \| * Chi square test for difference in frequency of postoperative cutaneous perforation between subgroups over all reconstruction techniques \| | | | | | | | | | | | | | | | |
